# A first principles study of a van der Waals heterostructure based on MS_2_ (M = Mo, W) and Janus CrSSe monolayers

**DOI:** 10.1039/d2na00298a

**Published:** 2022-07-12

**Authors:** Q. Alam, S. Sardar, H. U. Din, S. A. Khan, M. Idrees, B. Amin, F. Rehman, Saleh Muhammad, A. Laref

**Affiliations:** Department of Physics, Hazara University Mansehra KP Pakistan; Department of Physics, Bacha Khan University Charsadda KP Pakistan haleem.uddin@yahoo.com; Department of Physics, Abbottabad University of Science & Technology Havelian Abbottabad KP Pakistan binukhn@gmail.com; Department of Physics, Khushal Khan Khattak University Karak KP Pakistan; Department of Physics and Astronomy, College of Science, King Saud University Riyadh 11451 Saudi Arabia

## Abstract

The strategy of stacking two-dimensional materials for designing van der Waals heterostructures has gained tremendous attention in realizing innovative device applications in optoelectronics and renewable energy sources. Here, we performed the first principles calculations of the geometry, optoelectronic and photocatalytic performance of MS_2_–CrSSe (M = Mo, W) vdW heterostructures. The mirror asymmetry in the Janus CrSSe system allows the designing of two models of the MS_2_–CrSSe system by replacing S/Se atoms at opposite surfaces in CrSSe. The feasible configurations of both models of the MS_2_–CrSSe system are found energetically, dynamically and thermally stable. The studied heterobilayers possess an indirect type-I band alignment, indicating that the recombination of photogenerated electrons and holes in the CrSSe monolayer is hence crucial for photodetectors and laser applications. Remarkably, a red-shift in the optical absorption spectra of MS_2_–CrSSe makes them potential candidates for light harvesting applications. More interestingly, all heterobilayers (except W(Mo)S_2_–CrSSe of model-I(II)) reveal appropriate band edge positions of the oxidation and reduction potentials of the photocatalysis of water dissociation into H^+^/H_2_ and O_2_/H_2_O at pH = 0. These results shed light on the practical design of the MS_2_–CrSSe system for efficient optoelectronic and photocatalytic water splitting applications.

## Abstract 2

The strategy of stacking two-dimensional materials for designing van der Waals heterostructures has gained tremendous attention in realizing innovative device applications in optoelectronics and renewable energy sources.

## Graphical abstract

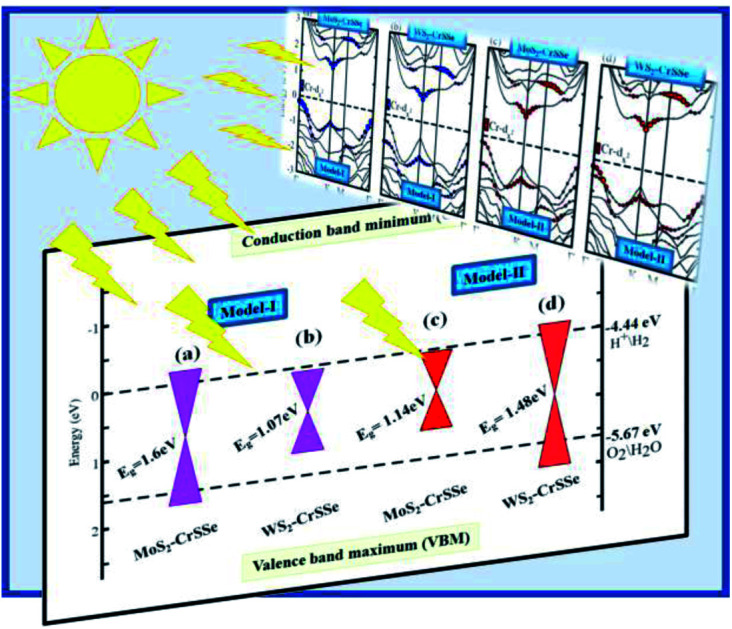

## Introduction

The isolation of single layer graphene has led to the emergence of novel two-dimensional (2D) materials for designing novel next-generation nanoelectronic and photonic devices.^1,2^ A majority of ultra-thin atomic layer materials such as hexagonal boron nitrides (h-BNs), transition metal dichalcogenide (TMDCs), and MXenes have been fabricated and widely studied due to their exceptional physical properties.^2–4^ In this sense, TMDCs with the general formula MX_2_ (M = Mo, W; X = S, Se) exhibit an extraordinary physical behavior compared to their bulk counterpart and have gained tremendous attention among the scientific community.^5^ The feasible fabrication, high thermal and mechanical structural stability, direct semiconducting band gap nature and unique optical properties of MX_2_ make them promising for optoelectronics,^6^ energy storage,^7^ gas sensing,^8^ field effect transistors,^9^ photocatalysis,^10^ and photovoltaic^11^ device applications.

In parallel with the search for new 2D materials, the formation of Janus configurations of TMDCs has been recently achieved as an alternative technique to tune the intrinsic behavior of already existing 2D TMDC systems. For instance, the Janus TMDC MoSSe monolayer has been practically realized through the chemical vapor deposition (CVD) technique by replacing sulfur with selenium in the MoS_2_ monolayer^12^ and replacing selenium with sulfur in the MoSe_2_ monolayer.^13^ Recently, Janus WSSe monolayers have been fabricated through the CVD method.^14^ The MXY (M = Mo, W; X, Y = S, Se) monolayers have proven excellent electronic and optical properties, realizing them potential candidates for designing optoelectronic,^15^ piezoelectric,^16^ photocatalysis,^17^ and spintronic devices.^18^ More recently, it has been demonstrated that all single layer Janus CrXY (X, Y = S, Se, Te) are energetically and thermally stable, and display semiconducting band gap nature (in the range of 1.10–1.57 eV), have good optical absorption in the near infra-red region and hold a suitable band edge for fully photocatalytic water splitting.^19^

The interest in tailoring the properties of 2D materials has gained tremendous attention in designing novel devices with enhanced tunable functionalities. The vertical assembling of 2D materials *via* weak van der Waals (vdW) interactions allows for the tailoring of the physical properties of the constituents, provides a feasible path for photogenerated charge separation and facilitates maximum optical absorption.^20^ The vdW heterostructures possess different types of band alignment. In the type-I band alignment, the valence band maximum (VBM) and conduction band minimum (CBM) are localized in one constituent, resulting in the fast recombination of photogenerated charge carriers and hence making them favorable for laser or light emitting diode (LED) applications.^21^ A type-II heterostructure holds both VBM and CBM localized in different constituents reducing the photogenerated charge separation, making them promising for solar cells and photovoltaics.^22^ Moreover, the continuous separation of photogenerated electrons and holes in different layers of the vdW heterojunction is utilized for water decomposition under solar irradiation.^23^

The photocatalytic water splitting reaction is driven by photoexcited electron–hole pairs, which are generated in a semiconducting photocatalyst as solar illumination and spatially separated by band bending at the semiconductor surface layer. Water at the photocatalyst surface can be reduced to H_2_ by photoexcited electrons that reach the surface, while holes at the surface would induce oxidation of water to produce O_2_ gas.^24–26^ Recently, several efforts have been dedicated to explore enhanced optoelectronic and photocatalytic properties in GeC–MXY,^27^ Janus TMDC–Janus TMDCs, MoSSe–WSSe,^28^ MoSSe–graphene,^29^ MoSSe–WSe_2_ (ref. 30) and MoSSe–XN.^31^ Despite these attempts, the theoretical or experimental study of a feasible design of the MS_2_–CrSSe vdW heterostructure remains unexplored.

In accordance with outstanding physical properties and satisfactory lattice mismatch, in the present study, we designed and investigated the unprecedented properties of the vdW heterostructure based on MS_2_ (M = Mo, W) and Janus CrSSe monolayers by first-principles calculations. The mirror asymmetry in the CrSSe monolayer allows for considering two different models of the MS_2_–CrSSe heterostructure with six possible stacking patterns. The most feasible stacking configuration of both models reveals energetic, dynamic and thermal stability. Finally, the electronic, optical and photocatalytic behavior of the feasible configuration is explored. Our findings predict MS_2_–CrSSe heterostructures as promising candidates for future optoelectronic and photovoltaic devices.

## Computational details

In present study, first principles calculations on the MS_2_–CrSSe heterostructure are performed using the projector augmented wave (PAW) method^32^ as implemented in the Vienna *ab initio* simulation package (VASP).^33–35^ The generalized gradient approximation (GGA) combined with the Perdew–Burke–Ernzerhof (PBE) functional^36^ with an energy cut-off of 600 eV was adopted to optimize the geometric structure and the HSE06 (Heyd–Scuseria–Ernzerhof) functional^37^ was used to correct the underestimated electronic band structures. The weak dispersion forces between the adjacent layers were described by the DFT-D2 scheme proposed by Grimme.^38^ A 6 × 6 × 1 Monkhorst–Pack *k*-point grid^39^ was used for geometric relaxation and further refined to 12 × 12 × 1 in the whole Brillouin zone (BZ) for the optimized geometry and electronic structure calculations. The interactions between the adjacent layers of the MS_2_–CrSSe heterostructure are avoided by using a vacuum slab of 25 Å along the *z*-axis. The convergence criteria of energy and force are set as 10^−6^ eV and 0.01 eV Å^−1^, respectively. The phonon band spectra are calculated using the density functional perturbation theory (DFPT) within the PHONOPY code.^40^*Ab initio* molecular dynamics (AIMD) calculations of the feasible structures are performed for a 6 × 6 × 1 supercell at room temperature. Bader charge analysis is adopted to demonstrate the charge transfers between the atom.^41,42^

## Results and dissection

The pristine single layers MS_2_ (M = Mo, W) and CrXY (X = S, Y = Se) exhibit a graphene-like hexagonal honeycomb structure. The calculated bond length for Mo–S, W–S, Cr–S and Cr–Se are 2.402 Å, 2.408 Å, 2.302 Å and 2.423 Å, respectively. Also, the optimized lattice constant (band gap) values for parent MoS_2_, WS_2_ and CrSSe monolayers are 3.16 Å (2.01 eV), 3.15 Å (1.95 eV) and 3.13 Å (2.11 eV), respectively. These results are consistent with previously available theoretical and experimental literature^43–51^ and confirm our theoretical approach for the study of TMDCs and Janus CrSSe monolayers.

**Fig. 1:**
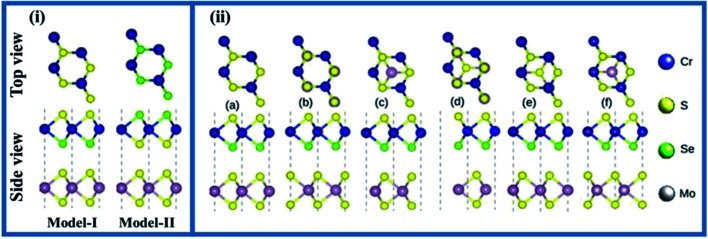
Side view and top view of (i) two models (ii) possible stacking patterns (a–f) of MS_2_–CrSSe (see text for details).

In general, the orientation of contacted atoms in individual layers or local configurations strongly affects the interfacial properties of the heterostructures. Since the lattice mismatch between Mo(W)S_2_ and CrSSe monolayers is 0.95 (0.63) %, the MS_2_ and CrSSe monolayers exhibit a satisfactory lattice mismatch, revealing the experimental construction of MS_2_–CrSSe heterostructures through van der Waals (vdW) interaction. As Janus CrSSe with mirror asymmetry possesses different chalcogen (X/Y) atoms, we consider two different models (model-I and model-II) with alternate chalcogen atoms at the opposite surface of single layer CrSSe placed above the MS_2_ monolayer to construct the MS_2_–CrSSe vdW-heterobilayer system, as displayed in Fig. 1(i). For each model, six possible configurations with different orientations of corresponding chalcogen or transition metal atoms in the MS_2_–CrSSe heterobilayer system are presented in Fig. 1(ii). In Fig. 1(ii); configuration a(b), the Se(Cr)-atom in CrSSe is localized on top of the S-atom in MoS_2_ and the Cr(Se)-atom occupies the position on top of the Mo-atom. Configuration c(d), Se-atom is positioned on top of the S(Mo)-atom with Mo(S)-atom localized on the hollow site. Configuration e(f), Cr-atom is located on top of Mo(S)-atom and S(Mo) occupies the hollow site. A similar trend of possible configurations with alternate orders of Se and S atoms in CrSSe is followed in model-II; hence, only model-I is discussed in detail here.

To further confirm the energetic stability of the most feasible configuration, the binding energy is calculated for each configuration of both models of MS_2_–CrSSe vdW heterostructures. The binding energy is defined as:*E*_b_ = *E*_hetero_ − *E*_TMDC_ − *E*_CrSSe_where *E*_b_ shows the binding energy of the system, *E*_hetero_, *E*_TMDC_ and *E*_CrSSe_ represent the total energy of MS_2_–CrSSe, isolated MS_2_ and CrSSe monolayers, respectively. The calculated lattice constants, bond length, binding energy (*E*_b_) and interlayer spacing (*d*) of the most feasible configuration for model-I and model-II are presented in Table 1. It is clear from Table 1 that an increase/decrease in the bond length of the Mo(W)S_2_/CrSSe system indicates intrinsic tensile/compressive strain in Mo(W)S_2_/CrSSe, respectively. The calculated intrinsic compressive/tensile strain are 0.47%/0.48% for MoS_2_/CrSSe in MoS_2_–CrSSe and 0.32%/0.32% for WS_2_/CrSSe in WS_2_–CrSSe, which is attributed to vdW interactions between Mo(W)S_2_ and CrSSe vertically stacked systems. Evidently, the disparity of local configurations leads to different magnitudes of binding energy and corresponding distance between the layers. The smaller interlayer spacing of the configuration (b) of both models reveals energetic stability and tailors the physical behavior of MS_2_ (M = Mo, W) and CrSSe monolayers.

**Table 1:** The optimized lattice constants (Å), bond length (Å), binding energy (*E*_b_, in eV) and interlayer spacing (*h*_spacing_, Å), band gap (PBE and HSE06 functionals, in eV), work function (eV), potential drop (eV) and band edge (for CBM and VBM) of the most feasible configuration of MS_2_–CrSSe systems for model-I (stacking-*a*) and model-II (stacking-*d*)

Heterostructure	Model-I		Model-II	
	MoS_2_–CrSSe	WS_2_–CrSSe	MoS_2_–CrSeS	WS_2_–CrSeS
*a* (Å)	3.145	3.14	3.145	3.14
*d*_Mo–S/W–S_ (Å)	2.40196	2.40815	2.40194	2.40816
*d*_Cr–Se_ (Å)	2.42303	2.42575	2.42312	2.42499
*d*_Cr–S_ (Å)	2.30146	2.30436	2.30	2.30375
*E*_(b)_ (eV)	−0.200	−0.253	−0.180	−0.283
*h*_spacing_ (Å)	3.2	3.0	3.1	3.0
*E*_g-PBE_ (eV)	0.60	0.31	0.32	0.65
*E*_g-HSE_ (eV)	1.60	1.07	1.14	1.48
*Φ* (eV)	3.79	3.39	3.59	3.79
Δ*V* (eV)	0.46	0.07	0.14	0.57
*E*_CBM_ (eV)	−0.23	0.13	0.01	−0.08
*E*_VBM_ (eV)	1.38	1.20	1.14	1.41

**Fig. 2:**
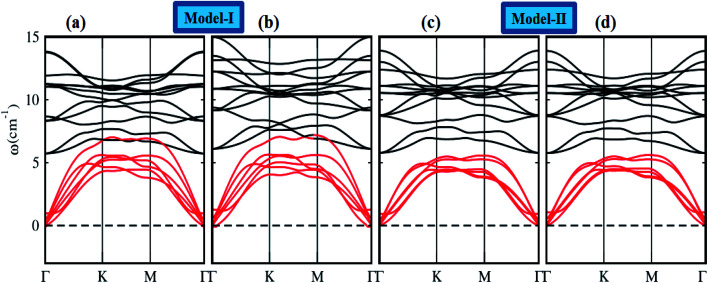
Phonon band spectra of (a and c) MoS_2_–CrSSe and (b and d) WS_2_–CrSSe heterostructures.

**Fig. 3:**
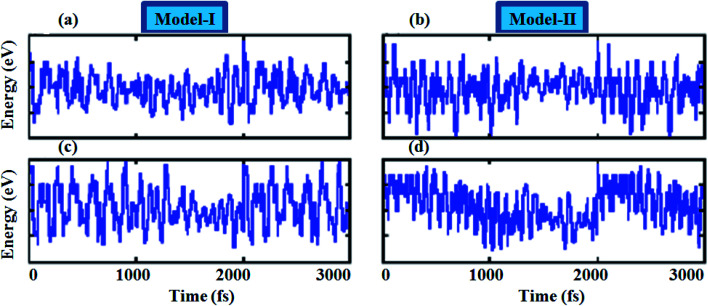
Thermal stability of (a and b) MoS_2_–CrSSe and (c and d) WS_2_–CrSSe using AIMD calculations.

To assess the dynamical stability of MS_2_–CrSSe heterobilayers, the phonon band spectra is calculated using the phonopy code, as shown in Fig. 2. There are six atoms per primitive unit cell therefore, each phonon band dispersion is composed of three acoustic zero frequency modes and 15 optical branches. As it is clear from Fig. 2 that all phonon modes with positive eigenfrequencies at the *Γ*-point of the Brillouin zone indicates dynamical stability of MS_2_–CrSSe heterobilayers. This confirms the synthesis of all MS_2_–CrSSe heterobilayers. Further, the thermal stability of a material is important to study the molecular vibrations or fluctuation in energy with time at different temperatures.^52,53^ The thermal stability of MS_2_–CrSSe systems, presented in Fig. 3(a–d), is carried out by performing *ab initio* molecular dynamics (AIMD) calculations. Since it determines the amount of energy per vibrational degree of freedom and the oscillation amplitudes are dependent on the temperature. Both models of MS_2_–CrSSe systems comprise pure harmonic oscillators, revealing no prominent change in the energy spectra, as shown in Fig. 3(a–d). This indicates the absence of broken bonds or the reconstruction of geometric structures after heating the system at 300 K for 3000 fs, hence confirming the thermodynamical stability of the MS_2_–CrSSe systems. This behavior has also been demonstrated in P–SiS, P–SiC and P-Janus structures.^54^

**Fig. 4:**
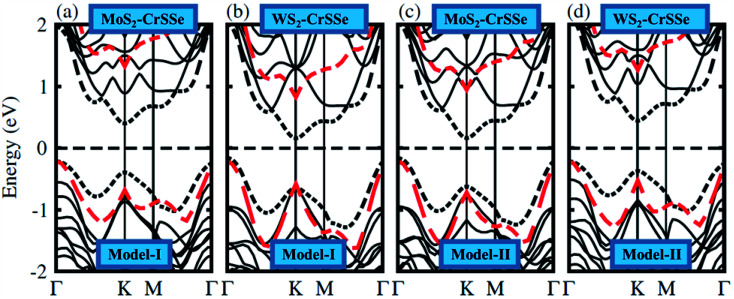
(a–d) Electronic band structures of MS_2_–CrSSe heterostructures and the black (red) dashed lines represent the PBE (HSE06) functional.

In general, the electronic properties of 2D materials can be modulated by the interaction between the stacked monolayers. The electronic band structure of MS_2_–CrSSe systems is investigated using PBE and HSE06 functionals, as displayed in Fig. 4(a–d). All understudy systems possess an indirect band gap semiconducting nature with the valence band maximum (VBM) located at *K*-point and the conduction band maximum (CBM) found at *Γ*-point of the Brillouin zone. In this case, the excited electrons could jump from the valence band to the conduction band at *K*-point and then the holes move from *Γ*-point, which can effectively retard the combination of photogenerated electrons and holes and enhance the photocatalytic performance^55^ TMDCs.^56,57^ This is consistent with other 2D In_2_X_3_ (X = S, Se, Te) monolayers^58^ and Nb_2_XTe_4_.^59^ The band gap values of MS_2_–CrSSe (model-I and model-II) systems using PBE and HSE06 functionals are listed in Table 1. Evidently, an enlarged band gap value using the HSE06 level can be seen. This trend has been demonstrated in GeC–MS_2_ and SiC–MX_2_ systems.^24,60,61^ Further, the MS_2_–CrSSe vdW heterostructures with indirect type-I band gaps are comparatively more promising for photodetectors and photocatalysis as compared to MX_2_–Zr_2_CO_2_,^62^ PbI_2_–α-Te^63^ and GaS–g-C_3_N_4_ (ref. 64) vdWs heterostructures.

**Fig. 5:**
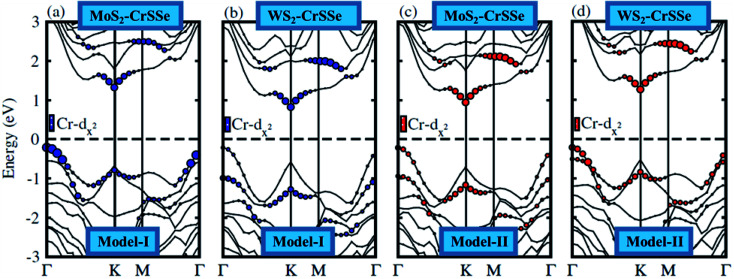
(a–d) Weighted band structure of MS_2_–CrSSe heterostructures.

In order to illuminate the orbital character for understanding the nature of the band alignment of heterostructures, the weighted band structure of all heterobilayers is calculated, as displayed in Fig. 5. For both models of MS_2_–CrSSe systems, VBM and CBM are mainly attributed by the Cr-d_*z*^2^_ state. It is evident from Fig. 5(a–d) that both VBM and CBM are localized in the CrSSe monolayer of the studied systems, indicating the type-I band alignment in MS_2_–CrSSe heterobilayers. The formation of the type-I band alignment is crucial for lasers or light emitting diodes (LEDs) applications.^65^ A similar trend of type-I band alignment has been theoretically demonstrated in ZnO–JTMDC, phosphorene–ZrSSe, and WSSe–WX_2_ (ref. 66–68) and experimentally reported in MoS_2_–PbI_2_.^69^

**Fig. 6:**
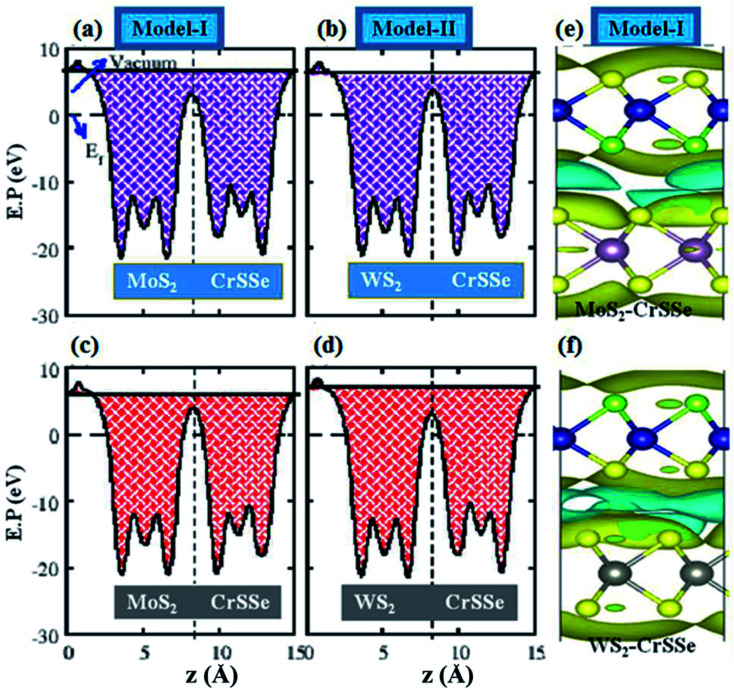
(a–d) Calculated plane averaged electrostatic potential (E.P) and (e and f) schematic representation of the charge density difference of MS_2_–CrSSe heterostructures, where the isovalue is chosen as 0.0025 e bohr^−3^.

The absence of mirror-symmetry in Janus 2D materials results in different polarization effects, which helps to understand the intrinsic electronic and extrinsic photocatalytic behavior of the materials.^70–73^ The plane-averaged electrostatic potential of all MS_2_–CrSSe heterobilayers for both models is calculated and plotted in Fig. 6(a–d). It can be clearly seen that all MS_2_–CrSSe heterobilayers have different potential energies with different positions of S- and Se-atoms in CrSSe in both models and results in different work functions (*Φ*) at S and Se atoms in CrSSe, as listed in Table 1. The calculated work function of understudy heterobilayers is comparatively smaller than the work functions of corresponding parent monolayers (5.15 eV (MoS_2_), 4.09 eV (WS_2_) and 5.24 eV (CrSSe) reported in ref. 23 and 38) and leads to strong interfacial interaction. In models-I, electrons may transfer from MoS_2_(WS_2_) with deeper potential to CrSSe with a lower potential, forming a built-in electric field at the interface with a potential drop of 0.46 eV(0.07 eV). Similarly, the potential drop (Δ*V*) for MoS_2_(WS_2_)–CrSeS is 0.14 eV(0.57 eV), which is consistent with C_4_N–MoS_2_.^74^ The built-in electric field formed at the interface moves from the MS_2_ (M = Mo, W) layer to the CrSSe layer for both model-I and model-II. Further, the charge density difference (Δ*ρ* = *ρ*_MS_2_–CrSSe_ − *ρ*_MS_2__ − *ρ*_CrSSe_) of the MS_2_–CrSSe system is calculated to address the interlayer charge transfer, as plotted in Fig. 6(e and f). Here, *ρ*_MS_2_–CrSSe_, *ρ*_MS_2__ and *ρ*_CrSSe_ represent the charge density of MS_2_–CrSSe, MS_2_ and CrSSe systems, respectively. In Fig. 6(e and f), the gain and loss of electrons are represented by yellow and cyan colors, respectively. It can be easily seen that MS_2_ depletes charges and the CrSSe layer accumulates charges. However, the main charge redistribution occurs in the interfacial region between chalcogen atoms of the individual layers, indicating a built-in electric field in the interfacial region. In both models, MS_2_–CrSSe heterobilayers exhibit the same character of charge transfer; hence, Δ*ρ* is plotted only for model-I of the Mo(W)S_2_–CrSSe system (Fig. 6(e and f)). This identifies that P-type doping occurs in MS_2_ monolayers. Moreover, the Bader charge analysis reveals that an amount of 0.0005*e* (0.0025*e*) is driven from MoS_2_ to CrSSe in model-I (model-II) and 0.0089*e* (0.0021*e*) is donated by WS_2_ to CrSSe in model-I (model-II). Interestingly, charge transfer in model-I (model-II) is responsible for stronger electronic influence in the WS_2_ (MoS_2_) monolayer.

**Fig. 7:**
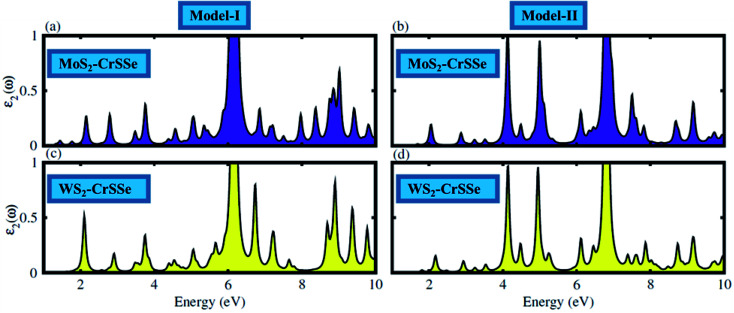
The absorption spectra of (a–d) MS_2_–CrSSe heterostructures.

The optical response of materials to incident solar light is crucial in identifying the promising materials for the water splitting mechanism. The optical parameter particularly the imaginary part of the dielectric function *ε*_2_(*ω*) of understudy systems is calculated to understand the transition between the occupied and unoccupied states, as displayed in Fig. 7(a–d). The electronic band structure is modified in the MS_2_–CrSSe system due to the weak coupling and interlayer charge transfer between the pristine monolayers. Moreover, the excitons dominate the optical transition from the valence band (VB) to the conduction band (CB) in all understudy heterostructures. Interestingly, the excitonic transition occurs in the range of 1.07–1.60 eV for MoS_2_–CrSSe, 2.05–2.80 eV for WS_2_–CrSSe, 2.01–2.80 eV for MoS_2_–CrSeS and 3.0–3.5 eV for WS_2_–CrSeS. In contrast to pristine monolayers, a red-shift is evidently observed in the optical absorption spectra of MS_2_–CrSSe, highlighting them as potential candidates for optoelectronic devices to capture the visible solar spectrum. Other 2D materials have followed the same trend.^75–78^

**Fig. 8:**
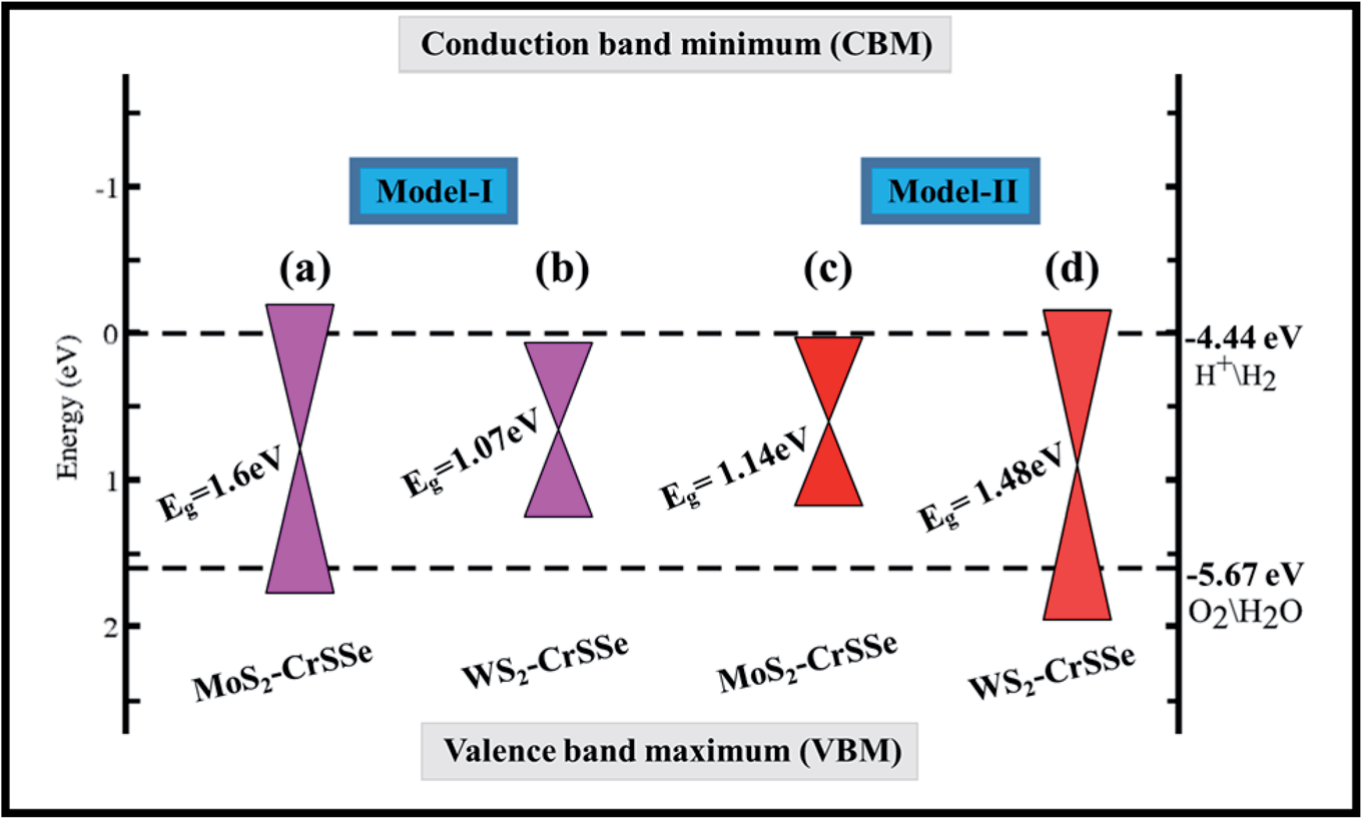
The conduction band and valence band edges of MS_2_–CrSSe systems for (a and b) model-I and (c and d) model-II. The black-dashed lines denote reduction (H^+^/H_2_) and oxidation (O_2_/H_2_O) potentials for water dissociation reactions.

To explore water dissociation into hydrogen production under solar irradiation, the valence band (VB) edge and conduction band (CB) edge position align with the standard reduction (H^+^/H_2_) and oxidation (O_2_/H_2_O) potentials of water dissociation are calculated at pH = 0 using the HSE06 level, as displayed in Fig. 8. In general, the standard potentials for the redox reactions of water splitting are as follows *E*_red_ (H^+^/H_2_) = −4.44 eV + pH × 0.059 eV and *E*_Ox_ (O_2_/H_2_O) = −5.67 eV + pH × 0.059 eV.^79^ In photocatalysis, photogenerated electrons and holes are separated and transported to H^+^/H_2_ or to O_2_ molecules. For the water splitting reaction, the photocatalyst should possess a semiconducting band gap with a value greater than 1.23 eV. Evidently, for W(Mo)S_2_–CrSSe of model-I(II), the valence band (VB) edge is found above the oxidation potential and the conduction band (CB) edge fails to reside above the reduction potential. In contrast, for other Mo(W)S_2_–CrSSe systems of model-I(II), the CBM lies higher than the reduction potential (−4.50 eV) and the VBM is located lower than the oxidation potential (−5.70 eV). This exciting trend of the band edge position has been demonstrated for other materials.^80,81^ Hence, both VB and CB edges straddle the redox potentials, rendering these systems as suitable candidates for producing low cost hydrogen gas at a commercial scale.

## Conclusion

To summarize, we theoretically investigated the structural, electronic, optical and photocatalytic behavior of two different models of MS_2_–CrSSe (M = Mo, W) heterostructures by performing the density functional theory calculations. The feasible binding energy, absence of imaginary frequencies in phonon branches and no considerable fluctuation of the energy curve at room temperature verify the energetic and dynamical stability of all understudy heterobilayers. All MS_2_–CrSSe (M = Mo, W) systems possess an indirect type-I band alignment with VBM and CBM localized in the CrSSe layer, indicating the recombination of photogenerated electrons and holes in the CrSSe layer, hence making them suitable for light detection applications. The broadening of light absorption in the visible region coupled with red-shift in absorption spectra reveals MS_2_–CrSSe systems promising for light conversion purposes. The Mo(W)S_2_–CrSSe of model-I(II) heterobilayers are capable of performing redox reactions of water splitting under solar irradiation, whereas the WS_2_(MoS_2_)–CrSSe system of model-I(II) fails to perform redox reactions. These findings lead to the practical utilization of MS_2_–CrSSe systems in future optoelectronic and photocatalytic water splitting applications.

## Conflicts of interest

The authors declare no conflict of interest.

## Supplementary Material
